# Immune Effective Score as a Predictor of Response to Neoadjuvant Trastuzumab Therapy and a Prognostic Indicator for HER2-Positive Breast Cancer

**DOI:** 10.3390/curroncol29010026

**Published:** 2022-01-10

**Authors:** Xueying Wu, Chenyang Zhang, Henghui Zhang

**Affiliations:** 1 Institute of Molecular Medicine, Peking University, Beijing 100871, China; 2101112232@pku.edu.cn (X.W.); 1801214236@pku.edu.cn (C.Z.); 2 Biomedical Innovation Center, Beijing Shijitan Hospital, School of Oncology, Capital Medical University, Beijing 100038, China

**Keywords:** HER2-positive breast cancer, neoadjuvant, trastuzumab, pathological complete response, prognosis

## Abstract

Background: HER2-positive breast cancer (BC) is a highly aggressive phenotype. The role of the host immune features in predictive response to anti-HER2 therapies and prognosis in BC has already been suggested. We aimed to develop a predictive and prognostic model and examine its relevance to the clinical outcomes of patients with HER2-positive BC. Methods: Immune effective score (IES) was constructed using principal component analysis algorithms. A bioinformatic analysis using four independent cohorts (GSE66305, *n* = 88; GSE130786, *n* = 110; TCGA, *n* = 123; METABRIC, *n* = 236) established associations between IES and clinical outcomes. Results: Genes associated with neoadjuvant trastuzumab therapy response were enriched in pathways related to antitumor immune activities. IES was demonstrated to be a predictive biomarker to neoadjuvant trastuzumab therapy benefits (GSE66305: area under the curve (AUC) = 0.804; GSE130786: AUC = 0.704). In addition, IES was identified as an independent prognostic factor for overall survival (OS) in the TCGA cohort (*p* = 0.036, hazard ratio (HR): 0.66, 95% confidence interval (CI): 0.449–0.97) and METABRIC cohort (*p* = 0.037, HR: 0.9, 95% CI: 0.81–0.99). Conclusion: IES has a predictive value for response to neoadjuvant trastuzumab therapy and independent prognostic value for HER2-positive breast cancer.

## 1. Introduction

Breast cancer (BC) with gene overexpression and/or amplification of Human Epidermal Growth Factor Receptor 2 (HER2) accounts for 15–20% of all BC cases [1]. Although HER2-positive BC is a highly aggressive phenotype, the emergence of trastuzumab and its application in the neoadjuvant therapy of early breast cancer has greatly enhanced the clinical outcomes of patients [2]. To date, the predictive biomarker for anti-HER2 agents is still HER2 overexpression or amplification. However, a number of such patients do not respond to HER2-targeted treatment in the neoadjuvant setting [3]. Potential predictive therapeutic biomarkers are therefore urgently needed to improve the efficacy of these therapies for HER2-positive BC patients.

Currently, there is growing evidence that patients with high levels of immune infiltration may benefit more from the anti-HER2 monoclonal antibody trastuzumab [4,5], indicating that tumor-infiltrating lymphocytes (TILs) might serve as a biomarker of anti-HER2 therapeutic response. On the other hand, HER2-positive BCs generally have higher TILs levels than luminal A/B BCs, implying that HER2-positive BCs are usually more immunogenic [6,7]. Therefore, immune characteristics are important prognostic indicators for HER2-positive BCs, as demonstrated in several studies [8]. Collectively, these findings indicate an important role for immunity in HER2-positive BC, both in the prediction of anti-HER2 therapeutic efficacy as well as the evaluation of prognosis. Nonetheless, the composition of the immune cell subsets is highly complex, and it is still unclear which immune effectors are functional for clinical outcomes in HER2-positive BC.

In the present study, significant immune signatures associated with the therapeutic response to anti-HER2 were identified based on the weighted correlation network analysis (WGCNA) method. We then established an immune effective score (IES) of 557 tumors in four cohorts from patients with confirmed HER2-positive BC. We found IES to be a predictive factor in response to neoadjuvant trastuzumab therapy and an independent prognostic biomarker.

## 2. Methods and Materials

### 2.1. Data Collection

Single nucleotide variation (SNV) and copy number variation (CNV) data of TCGA-BRCA were obtained from the GDC Data Portal (https://portal.gdc.cancer.gov/, accessed on 3 August 2020). The RNAseq data and clinical information of the TCGA-BRCA cohort were obtained from UCSC Xena (https://xena.ucsc.edu/, accessed on 3 August 2020). Microarray data were obtained from GEO (https://www.ncbi.nlm.nih.gov/geo/query/, GSE66305, GSE130786, accessed on 1 July 2020). Gene expression data and clinical information of the METABRIC cohort were downloaded from the cBioPortal (http://www.cbioportal.org/, accessed on 21 August 2020). Patients enrolled in this study should have HER-2 positive status, which was confirmed by immunohistochemistry (IHC). Thus, a total of 557 women (GSE66305, *n* = 88; GSE130786, *n* = 110; TCGA, *n* = 123; METABRIC, *n* = 236) were included based on this criterion. The detailed workflow is shown in Figure S1.

### 2.2. Weighted Gene Coexpression Network Analysis

The gene coexpression networks were constructed by the WGCNA package [9] in R (version 3.6.3). A coexpression network was constructed by 5044 genes with variance ranked in the top 25%, and the Pearson correlations were calculated between all genes. Based on the network’s scale-free topology, a β (soft-thresholding power) parameter was determined to reconstruct the network for strongly correlated genes and to exclude weakly correlated genes.

### 2.3. Immune-Related Signature Analysis

The gene sets for CD8 T cells, Tfh, Th1, NK, cytolytic activity, inflammation promoting and T-cell costimulation were used in a previous study [10]. Each signature score was calculated by single-sample gene-set enrichment analysis (ssGSEA) using “GSVA” package (method = “ssgsea”) in R (version 3.6.3).

### 2.4. Gene-Set Enrichment Analysis (GSEA)

Gene-set enrichment analysis (GSEA) was performed using the GSEA software provided by the Broad Institute. Reactome pathway gene sets were obtained from MSigDB version 7.1 (http://software.broadinstitute.org/gsea/msigdb, accessed on 1 October 2019).

### 2.5. Statistical Analysis

All statistical analyses were performed using R version 3.6.3 software (Institute for Statistics and Mathematics, Vienna, Austria; www.r-project.org, accessed on 19 August 2019). Correlations were tested with Spearman’s rank correlation test. The Wilcoxon test was used to compare two groups of continuous variables. Fisher’s exact test was applied for comparisons between two categorical variables. Survival analysis was performed using a Kaplan–Meier survival plot, and the log-rank test *p*-value was calculated. The univariate and multivariate prognosis analyses were estimated by a multivariate Cox proportional hazards regression model. *p* values were corrected using Benjamini–Hochberg FDR methods. Statistical significance was set at *p* < 0.05.

## 3. Results

### 3.1. Genes Associated with Neoadjuvant Trastuzumab Therapy Response Are Enriched in Antitumor Immune Signaling Pathways

**Figure 1:**
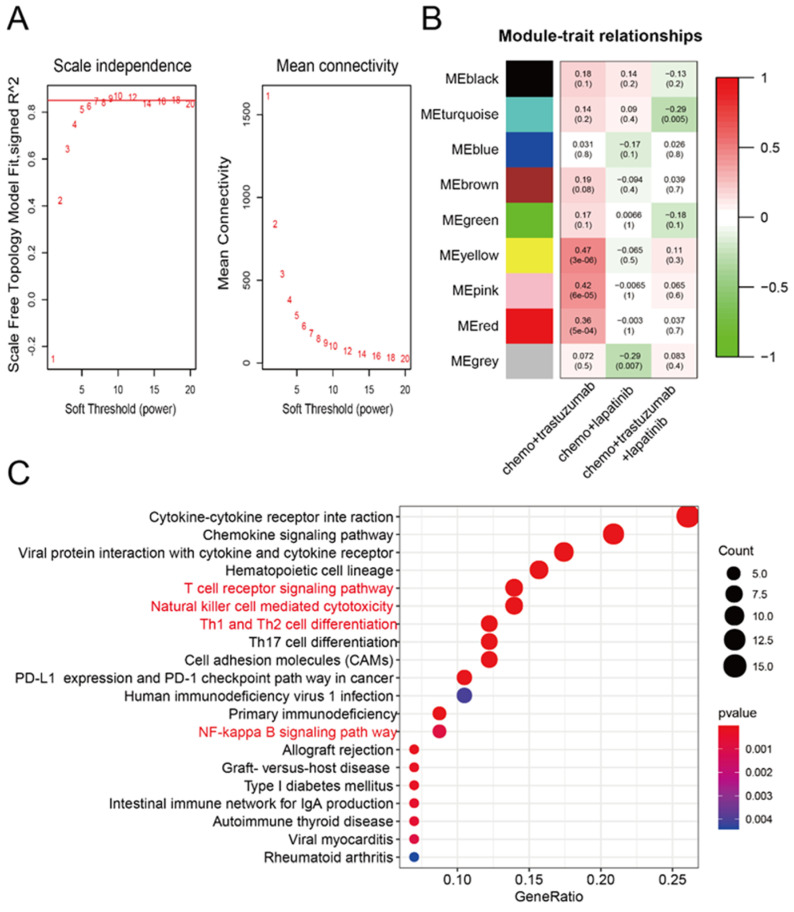
Construction of the gene coexpression network from patients exposed to neoadjuvant anti-HER2 therapies. (**A**) Selection of the soft-thresholding powers. The left panel shows the scale-free fit index versus the soft-thresholding power. The right panel displays the mean connectivity versus soft-thresholding power. The digital numbers stand for different β (soft-thresholding power) parameter and the red horizontal line represents the cutoff (0.9) of scale-free topology index. (**B**) Module-trait relationships. Each row represents a ME, the three columns represent the response to chemo + trastuzumab, chemo + lapatinib, and chemo + trastuzumab + lapatinib, respectively, and each cell contains the corresponding correlation and *p*-value. The matrix is color-coded by correlation according to the color legend. Chemo: chemotherapy. (**C**) KEGG enrichment analysis of coexpression modules identified by WGCNA. KEGG: the Kyoto Encyclopedia of Genes and Genomes. The y-axis represents the number of gene counts in the KEGG term. The intensity and color of dots are indicated on the right side of the heatmap and are represented by their corresponding *p*-values.

To identify genes related to neoadjuvant trastuzumab therapy response, we first constructed a coexpression network using samples from 88 patients with breast cancer who had undergone different neoadjuvant therapies (GSE66305). WGCNA was used with a screen-out power from 1 to 20, and *β* = 10 was selected as the best value for the soft threshold of the network, resulting in a scale-free topology index (*R^2^*) of 0.9 and higher average connectivity (Figure 1A). A total of nine coexpression modules were constructed and are shown in different colors, and the module–trait heatmap is shown in Figure 1B. The yellow, pink and red modules all had a high correlation with response to neoadjuvant trastuzumab therapy (all *R*  >  0.35, *p* < 0.001), whereas for other therapies and modules no correlation was observed (Figure 1B). We then performed enrichment analysis on the Kyoto Encyclopedia of Genes and Genomes (KEGG) to categorize the biological functions of genes found in these three modules using the “enrichKEGG” programming function in R’s “clusterProfiler” package [11]. Interestingly, our data showed that genes associated with neoadjuvant trastuzumab therapy response were highly enriched in pathways closely related to antitumor immune activities, including the T-cell receptor signaling pathway, natural killer cell-mediated cytotoxicity and NF−kappa B signaling pathway (Figure 1C).

### 3.2. Construction of Immune Effective Score (IES)

**Figure 2:**
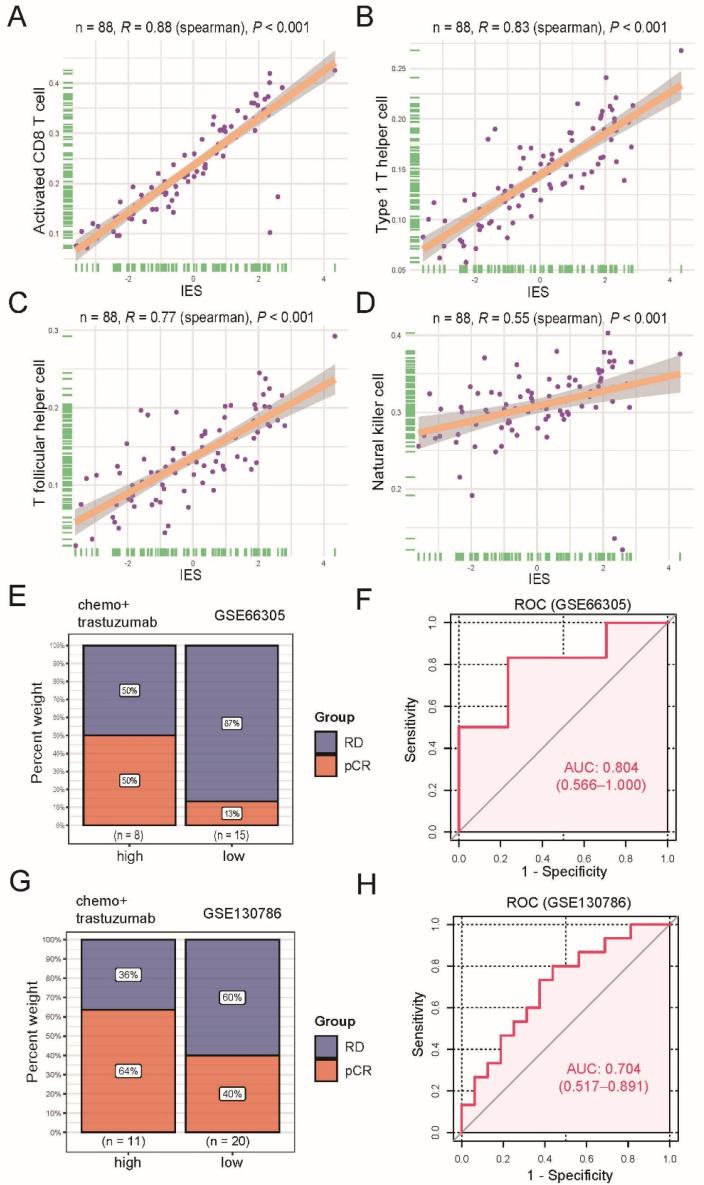
Functional and predictive analysis of immune effective score (IES). Scatter plots display the positive correlation between IES and activated CD8 T cell (**A**), type 1 T helper cell (**B**), T follicular helper cell (**C**), natural killer cell (**D**). The correlation coefficient (*R*) and *p* values calculated by Spearman’s rank correlation test are given in the figure. The dots represent the data, and the solid line represents the best-fitting curve. The shades around fitted curves indicate 95% confidence interval. Bar plots depict the clinical response rate to neoadjuvant trastuzumab therapy in high or low IES groups in the GSE66305 cohort (**E**) and GSE130786 cohort (**G**). pCR: complete response; RD: residual disease. Receiver operating characteristic (ROC) analysis for predicting pCR to neoadjuvant trastuzumab therapy in the GSE66305 cohort (**F**) and GSE130786 cohort (**H**). Area under the curve (AUC) values are marked and the corresponding 95% confidence interval (CI) is provided in the parentheses.

Previous research has shown that cytotoxic CD8 T cells [12,13], T helper type 1 (Th1) cells [14]), follicular helper T (Tfh) cells [15] and natural killer (NK) cells [16] were strongly associated with patient survival or response to therapy in BC. These reports, combined with our data, suggest that immune effective signatures may contribute to trastuzumab’s beneficial therapeutic effects. Therefore, seven signatures which have been reported for the classification of triple-negative breast cancers (TNBC) immune phenotypes [10], including CD8 T cells, Tfh, Th1, NK, cytolytic activity, inflammation promoting and T-cell co-stimulation, were chosen for further analysis (see Table S1 for all gene sets). On the basis of the ssGSEA scores of the seven signatures, a principal component analysis (PCA) was performed, and immune effective score (IES) of each patient was defined as the sum of the first two principal components. To test the accuracy of IES, we further calculated the level of tumor-infiltrating immune cells (TILs) using a previously published method [17], and examined the correlations of the IES and TILs. A statistically significant positive correlation between IES and infiltration of activated CD8 T cells (*Spearman R* = 0.88, *p* < 0.001; Figure 2A), Th1 (*Spearman R* = 0.83, *p* < 0.001; Figure 2B), Tfh (*Spearman R* = 0.77, *p* < 0.001; Figure 2C) and NK cells (*Spearman R* = 0.55, *p* < 0.001; Figure 2D) was seen, suggesting IES could serve as a signature score, as expected.

### 3.3. The IES Predicts Neoadjuvant Trastuzumab Therapy Benefits

Next, two datasets (GSE66305 and GSE130786) were used for investigating whether there was an association between IES at baseline and pathological complete response (pCR) rates of neoadjuvant trastuzumab therapy. The top one-third of the IES value was used as the cutoff for identifying a high-IES tumor. Results showed a higher pCR rate in the GSE66305 cohort high-IES community (50% vs. 13%; Figure 2E) and the GSE130786 cohort (64% vs. 40%; Figure 2G) compared with the corresponding low-IES groups. However, we did not observe a similar trend with other neoadjuvant therapies (Figure S2). Moreover, when the IES was evaluated as a continuous variable with the receiver operating characteristic (ROC) analyses, IES was also demonstrated to be a predictive biomarker of neoadjuvant trastuzumab therapy benefits (GSE66305: area under the curve (AUC) = 0.804; Figure 2F; GSE130786: AUC = 0.704; Figure 2H).

### 3.4. IES Is Associated with FGFR1 Signaling in HER2-Positive BC

**Figure 3:**
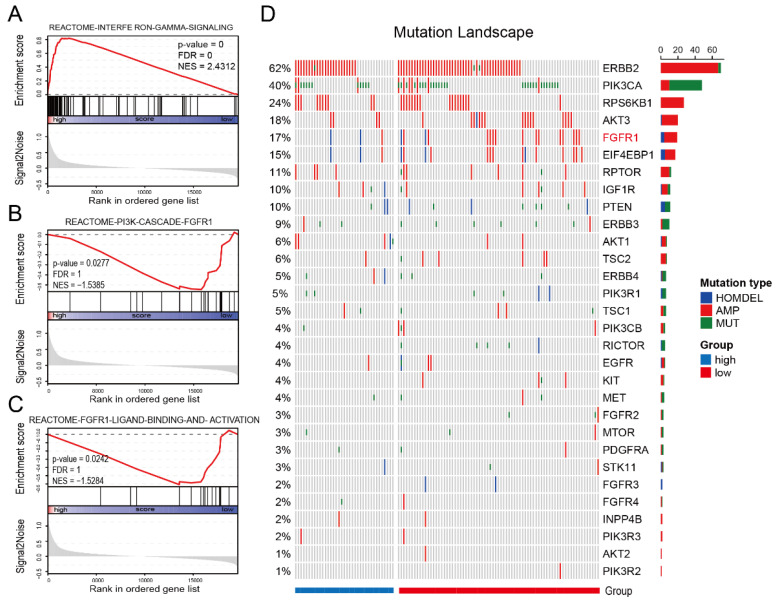
Low-IES status is associated with FGFR1 signaling. Enrichment plots from gene-set enrichment analysis (GSEA) for the interferon-gamma signaling (**A**), PI3K cascade FGFR1 signaling (**B**) and FGFR1 ligand binding and activation signaling (**C**). The red lines indicate normalized enrichment scores (NES) calculated by GSEA. The height of the bars indicates the running GSEA enrichment score. Mutational landscape of PI3K pathway-related genes in the TCGA cohort (**D**). The column and row represent patients and genes, respectively. The patients are displayed in a descending order based on the number of mutated genes. The right panel indicates the frequency of gene mutations. Different colors indicate different types of mutations. Gray denotes no mutations. HOMDEL: homozygous deletion; AMP: amplification; MUT: mutation.

In addition to immune profiles, trastuzumab resistance is also correlated with genomic changes such as mutations in *PI3KCA* [18], *PTEN* loss [19], and HER2 dimerization partners amplification [20]. Hence, we further utilized gene expression data, simple nucleotide variation (SNV), and copy number variation (CNV) information on HER2-positive BC patients in TCGA (*n* = 123) to identify biological processes linked to IES status. Patients in the TCGA cohort were classified into low and high (-IES) groups as previously described, and GSEA analysis was carried out to identify the IES associated with the biological signaling pathway. Since interferon-γ can be produced by the CD8 T cells, Th1, Tfh and NK cells, it was no surprise that interferon-gamma signature genes were significantly enriched in high-IES samples (normalized enrichment scores (NES) = 2.4312; Figure 3A). However, it is worth noting that the FGFR–PI3K signaling axis was enriched in the low-IES group (NES = −1.5385; Figure 3B; NES = −1.5284; Figure 3C). The mutational landscape of PI3K pathway-related genes showed that FGFR1 amplification was observed in only 2.5% (1/40) of high-IES samples, but 17.3% (14/81) of low-IES samples (Figure 3D). This implies that trastuzumab resistance in low-IES patients may be related to activation of the FGFR1 signaling pathway.

### 3.5. Evaluation of IES for the Prognostic Prediction of HER2-Positive BC in TCGA Cohort and METABRIC Cohort

**Figure 4:**
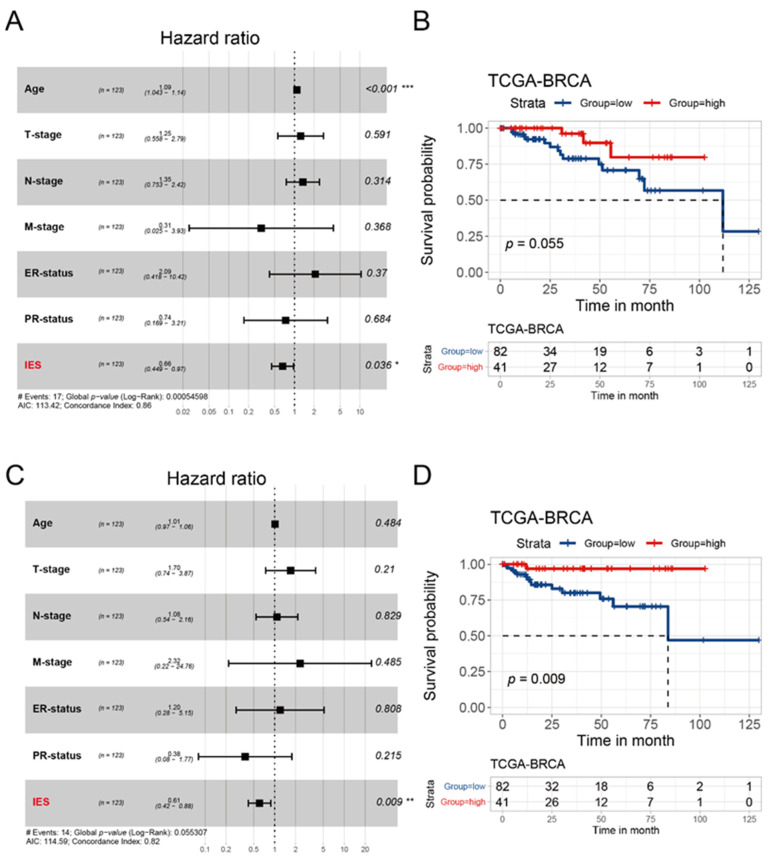
Prognostic analysis of the IES in the TCGA cohort. Forest plots of multivariate Cox proportional hazards regression analysis of OS (**A**) and PFI (**C**) in the TCGA cohort. The *p* values was calculated by Cox regression (* *p* < 0.05, ** *p* < 0.01, *** *p* < 0.001). OS: overall survival; PFI: progression-free interval. Kaplan–Meier survival curves show the OS (**B**) and PFI (**D**) stratified by low/high-IES in the TCGA cohort. The *p* values were obtained by the log-rank test.

From a variety of studies, it is now well-established that immune functions play an important prognostic role in BC [21,22,23]. Thus, we sought to explore whether the IES as a predictor in HER2-positive BC has prognostic potential. Clinical information data were collected from patients with confirmed HER2-positive status in the TCGA cohort (*n* = 123) and METABRIC cohort (*n* = 236). After multivariate Cox regression analysis of all relating factors, IES was identified as an independent prognostic factor for overall survival (OS) in the TCGA cohort (*p* = 0.036, hazard ratio (HR): 0.66, 95% confidence interval (CI): 0.449–0.97; Figure 4A) and METABRIC cohort (*p* = 0.037, HR: 0.9, 95% CI: 0.81–0.99; Figure S3A). As a grouping variable, there was a trend toward improvement in the OS for high-IES patients from the TCGA cohort (*p* = 0.055; Figure 2B) and METABRIC cohort (*p* = 0.065; Figure S3B). Consistent with the OS results, IES (*p* = 0.009, HR: 0.61, 95% CI: 0.42–0.88; Figure 4C) was also independently linked to progression-free interval (PFI) in the multivariate Cox regression analysis. Besides, patients assigned to the high-IES group had significantly better PFI than those who were assigned to the low-IES group (*p* = 0.009; Figure 4D). In summary, our data suggest that the IES is independent of conventional clinical characteristics and performed well in survival prediction for HER2-positive BC.

## 4. Discussion

Previous studies have noted the strong relationship between the host immune response and neoadjuvant trastuzumab therapy of HER2-positive BC patients [4,5]. Researchers often use a single immune-related gene [24] or overall immune infiltration [8] as a predictive biomarker for neoadjuvant trastuzumab therapy. Although these results have important implications, they may not systematically indicate an effector immune response in patients. In this study, we found that genes associated with response to neoadjuvant trastuzumab therapy were highly enriched in pathways closely related to antitumor immune activities. Interestingly, most of these pathways including cytotoxic CD8 T cells, Th1, Tfh and NK signatures were important in the production of IFN-γ. Hence, based on the above conclusion, we designed an IES to evaluate the immune effective phenotype. In two independent cohorts, high-IES patients showed a higher pCR rate for preoperative trastuzumab therapy than the low-IES cohort.

Several anti-HER2 agents are currently used in the neoadjuvant setting, including trastuzumab, pertuzumab and lapatinib [25,26]. The combination of dual anti-HER2 blockade, such as trastuzumab plus lapatinib and trastuzumab plus pertuzumab, has shown increased effectiveness over single blockade in clinical studies (NeoALTTO trial, NCT00553358 [27]; CHER-LOB trial, NCT00429299 [28]; NeoSphere trial, NCT00545688 [29]). However, trastuzumab alone remains the standard of care in combination with systemic chemotherapy (paclitaxel-based), given the possibility of increased toxicity. A recent review showed a range of pCR rate between 29% and 52% for neoadjuvant trastuzumab therapy [2]. Due to the different chemotherapy regimens, as well as the inclusion/exclusion criteria, it is difficult to compare pCR levels between different studies. What is interesting about our data is the pCR rate from the GSE66305 cohort in the high-IES group (88 samples from CHER-LOB trial) reached 50%, but the overall pCR rate in chemo + trastuzumab arm was only 25% in CHER-LOB trial [28]. In the GSE66305 cohort, patients from the high-IES group in chemo + trastuzumab arm also showed a higher pCR rate (64%) than the average level previously reported. No similar results were observed in arms with other treatment measures, implying that IES may serve as a predictive biomarker specific to neoadjuvant trastuzumab therapy.

Clinically, although pCR is now a surrogate endpoint in neoadjuvant trials with regulatory intent endorsed by FDA, the long-term survival and the quality of life of patients are still the biggest concern for clinicians. Unfortunately, we were short of follow-up information for GSE66305 and GSE130786. Thankfully, we found recent studies on survival analyses of the relevant trials, and the strong association between pCR and long-term survival or recurrence is maintained in all these randomized clinical trials [30,31,32]. In addition, the subgroup analysis of the I-SPY2 trial showed that the pCR of the trastuzumab treatment arm was more significantly correlated with the long-term survival of patients [33]. Therefore, combined with our data, patients with higher IES may derive significant survival benefits due to easier access to pCR from trastuzumab neoadjuvant treatment. Another issue that needs to be considered is that women who undergo breast conservation have reported higher satisfaction with body image than women who undergo mastectomy. It has been reported that the rate of breast conservation in patients who achieved pCR in the breast was much higher than in those with RD [34]. However, whether to obtain the possibility of breast-conserving surgery (BCS) through neoadjuvant therapy is a gamble for some high-risk patients. It means that patients may receive overtreatment once pCR is not achieved. Thus, it would be advantageous if the pCR could be predicted in advance. Additionally, we hope IES could help doctors decide whether to perform neoadjuvant treatment for these high-risk patients who have strong willingness to achieve breast conservation.

Interestingly, based on TCGA cohort analysis we found an enrichment in the low-IES community for activation of the FGFR1–PI3K–mTOR signaling axis. FGFR1, as a member of transmembrane receptor tyrosine kinase (RTKs), has been reported to be associated with resistance to anti-HER2 therapies [20,35]. These findings suggest that in combination with trastuzumab, patients in the low-IES group can derive more benefit from FGFR inhibitor therapies. However, FGFR inhibitors have not yet reached clinical routine practice in BC and therefore these results should be interpreted with caution.

In addition, high rates of lymphocyte infiltration have been linked with a more favorable prognosis in patients with triple-negative and HER2-positive breast cancer [36]. This is an indication that the immune-effective phenotype may also be associated with a better prognosis. We applied multivariate Cox regression analysis to the TCGA cohort and METABRIC cohort to discern this property. Further, we demonstrated the significance of the IES in predicting survival in HER2-positive breast cancer. Besides, the PFI benefit was much more pronounced, indicating that patients with low-IES are prone to progression and recurrence.

This study has several limitations. First, we did not have any survival information for the GEO cohorts available to explore the prognostic value of IES for disease-free survival (DFS) and event-free survival (EFS) in this study. Second, there is a need for more validation cohorts to verify the prediction and prognostic validity of the IES. These limitations mean that the study findings should be interpreted with caution, and further studies are required.

## 5. Conclusions

Taken together, we constructed an IES which has predictive value for response to neoadjuvant trastuzumab therapy and independent prognostic value for HER2-positive breast cancer.

## Data Availability

All data are openly available in public databases. Single nucleotide variation (SNV) and copy number variation (CNV) data of TCGA-BRCA are openly available in the GDC Data Portal (https://portal.gdc.cancer.gov/, accessed on 3 August 2020). The RNAseq data and clinical information of TCGA-BRCA cohort are openly available in UCSC Xena (https://xena.ucsc.edu/, accessed on 3 August 2020). Microarray data are openly available in GEO (https://www.ncbi.nlm.nih.gov/geo/query/ (accessed on 1 July 2020), GSE66305, GSE130786). Gene expression data and clinical information of METABRIC cohort are openly available in the cBioPortal (http://www.cbioportal.org/, accessed on 21 August 2020).

## Supplementary Materials

The following are available online at https://www.mdpi.com/article/10.3390/curroncol29010026/s1. Figure S1: Workflow diagram., Figure S2: Predictive analysis of IES, Figure S3: Prognostic analysis of the IES in the METABRIC cohort. Table S1. Immune signature gene sets.

## Abbreviations

AUC	area under the curve
BC	breast cancer
CI	confidence interval
GEO	gene expression omnibus database
HR	hazard ratio
IES	immune effective score
KEGG	Kyoto Encyclopedia of Genes and Genomes
NK	natural killer cells
OS	overall survival
PCA	principal component analysis
pCR	pathological complete response
PFI	progression-free interval
RD	residual disease
ROC	receiver operating characteristic
ssGSEA	single-sample gene-set enrichment analysis
TCGA	the cancer genome atlas
Tfh	follicular helper T cells
Th1	T helper type 1 cells
TILs	tumor-infiltrating lymphocytes
TNBC	triple-negative breast cancer
WGCNA	weighted correlation network analysis
