# Supplementary material for: Immune Effective Score as a Predictor of Response to Neoadjuvant Trastuzumab Therapy and a Prognostic Indicator for HER2-Positive Breast Cancer
Source: Curr Oncol. 2022 Jan 10;29(1):283–93. doi: 10.3390/curroncol29010026 (PMC8775173; doi:10.3390/curroncol29010026)
Supplement: Supplementary file 1 [file curroncol-29-00026-s001.zip › curroncol-1443172-supplementary.pdf]

Current Oncology

Supplementary Materials

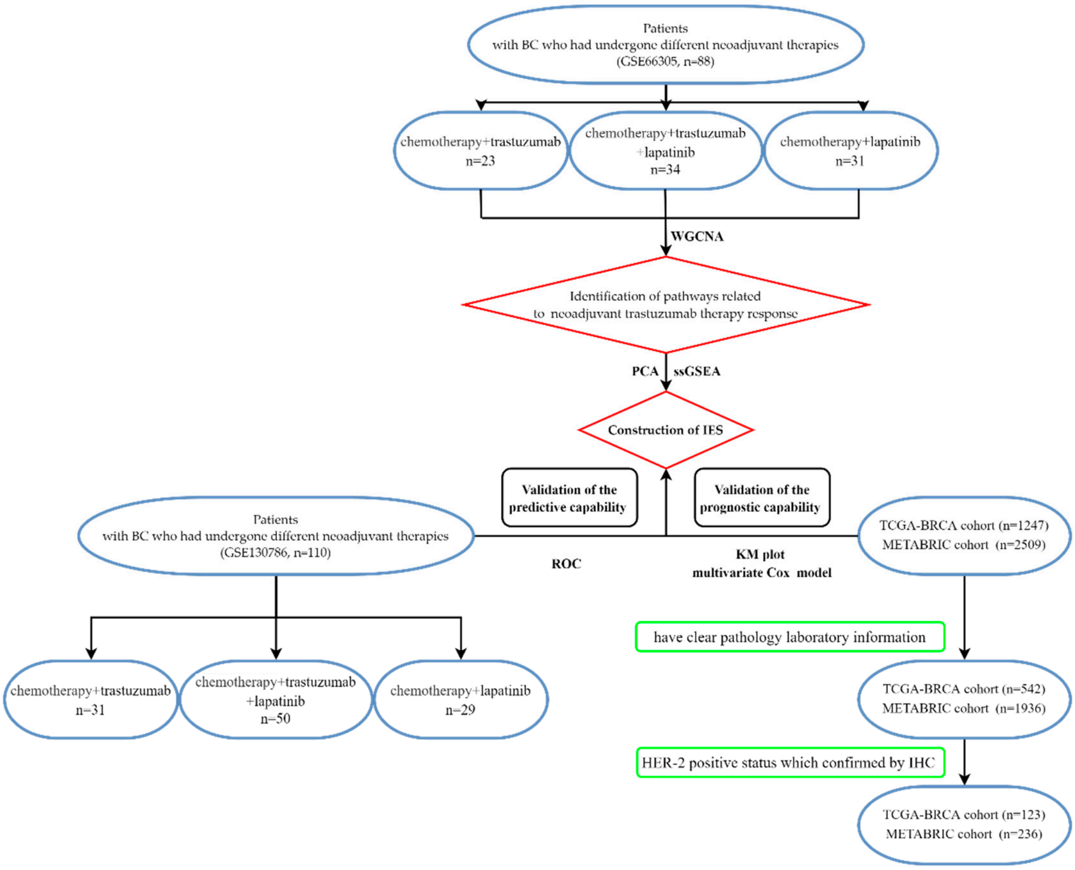

Figure S1. Work flow diagram. BC: breast cancer; WGCNA: weighted correlation network analysis; PCA: principal component analysis; ssGSEA: single-sample gene set enrichment analysis; IES: immune effective score; ROC: receiver operating characteristic; KM plot: Kaplan-Meier survival plot.

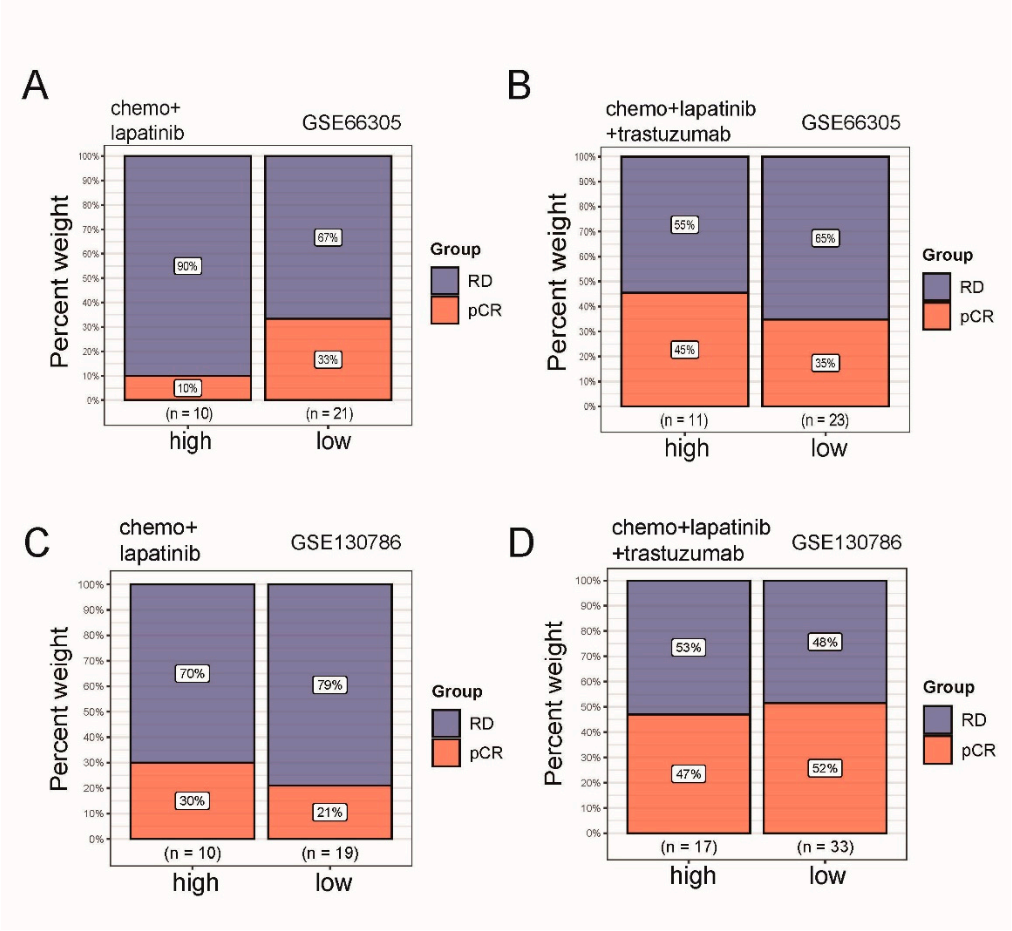

Figure S2. Predictive analysis of IES. Bar plots depict the clinical response rate to neoadjuvant chemo + lapatinib therapy in high or low IES groups in the GSE66305 cohort (**A**) and GSE130786 cohort (**C**). Bar plots depict the clinical response rate to neoadjuvant chemo + laptinib + trastuzumab therapy in high or low IES groups in the GSE66305 cohort (B) and GSE130786 cohort (**D**). pCR: complete response; RD: residual disease.

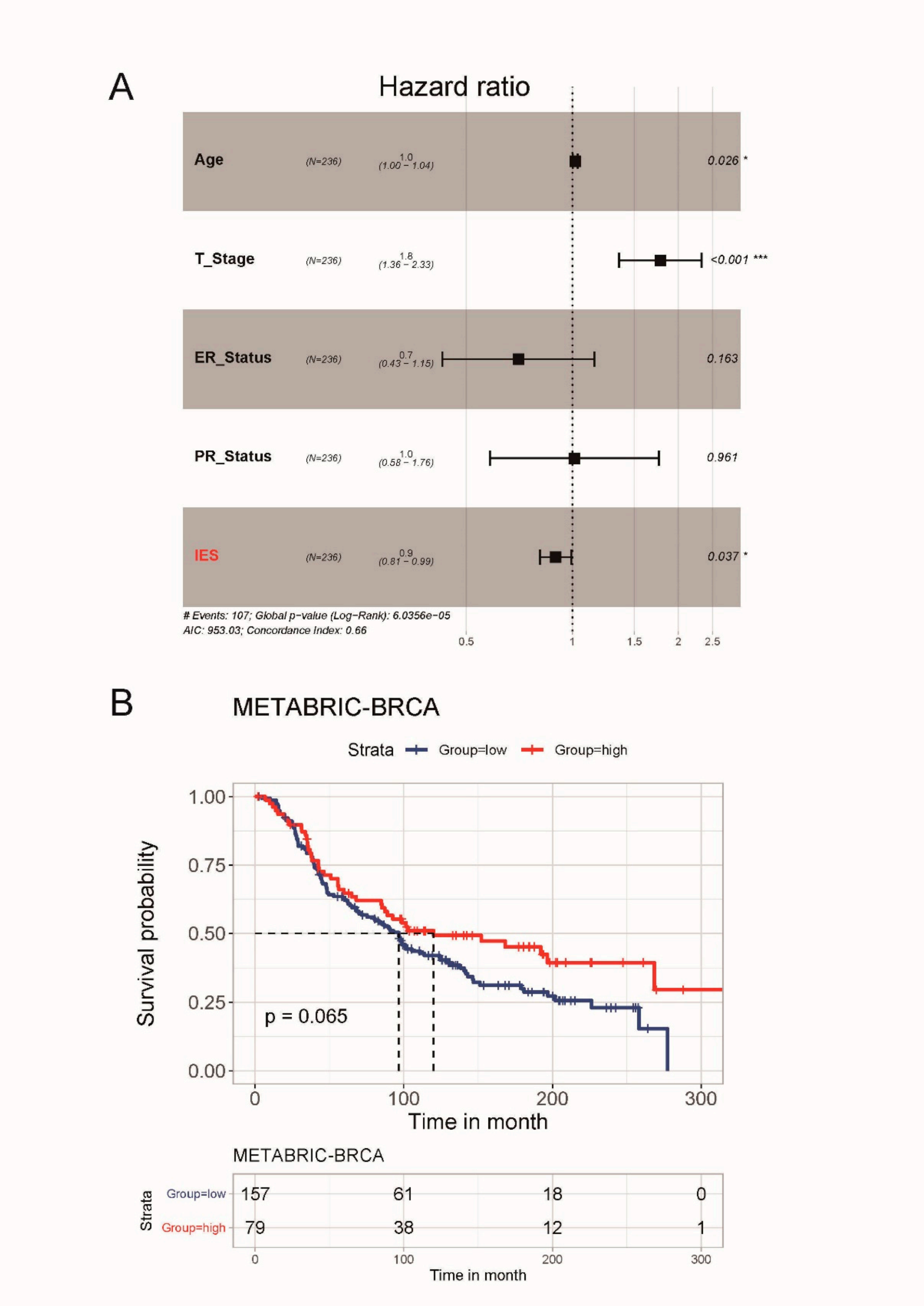

Figure S3. Prognostic analysis of the IES in the METABRIC cohort. Forest plots of multivariate Cox regression proportional hazards regression analysis of OS in the METABRIC cohort (**A**). *P*-value was calculated by cox regression (* $P < 0.05$, ** $P < 0.01$, *** $P < 0.001$). OS: overall survival. Kaplan-Meier survival curves showed the OS stratified by low/high-IES in the METABRIC cohort (**B**). *P* values were obtained by the log-rank test.

Table S1. Immune signature gene sets.

CD8 T cells	Cytolytic activity	Inflammation-promoting	NK cells	T cell co-stimulation	Tfh	Th1 cells
CD8A	PRF1	CCL5	KLRC1	CD2	PDCD1	IFNG
	GZMA	CD19	KLRF1	CD226	CXCL13	TBX21
		CD8B		CD27	CXCR5	CTLA4
		CXCL10		CD28		STAT4
		CXCL13		CD40LG		CD38
		CXCL9		ICOS		IL12RB2
		GNLY		SLAMF1		LTA
		GZMB		TNFRSF18		CSF2
		IFNG		TNFRSF25		
		IL12A		TNFRSF4		
		IL12B		TNFRSF8		
		IRF1		TNFRSF9		
		PRF1		TNFSF14		
		STAT1				
		TBX21				
